# Alterations of Elastic Property of Spastic Muscle With Its Joint Resistance Evaluated From Shear Wave Elastography and Biomechanical Model

**DOI:** 10.3389/fneur.2019.00736

**Published:** 2019-07-10

**Authors:** Yan Leng, Zhu Wang, Ruihao Bian, Wai Leung Ambrose Lo, Xiaoyan Xie, Ruoli Wang, Dongfeng Huang, Le Li

**Affiliations:** ^1^Department of Rehabilitation Medicine, The First Affiliated Hospital, Sun Yat-sen University, Guangzhou, China; ^2^Department of Medical Ultrasonics, Institute of Diagnostic and Interventional Ultrasound, The First Affiliated Hospital, Sun Yat-sen University, Guangzhou, China; ^3^Department of Women's and Children's Health, Karolinska Institutet, Stockholm, Sweden; ^4^Department of Mechanics, Royal Institute of Technology, Stockholm, Sweden; ^5^KTH BioMEx Center, Royal Institute of Technology, Stockholm, Sweden; ^6^Department of Rehabilitation Medicine, The Seventh Affiliated Hospital, Sun Yat-sen University, Shenzhen, China

**Keywords:** rehabilitation, stroke, spasticity, muscle, shear wave elastography

## Abstract

This study aims to quantify passive muscle stiffness of spastic wrist flexors in stroke survivors using shear wave elastography (SWE) and to correlate with neural and non-neural contributors estimated from a biomechanical model to hyper-resistance measured during passive wrist extension. Fifteen hemiplegic individuals after stroke with Modified Ashworth Scale (MAS) score larger than one were recruited. SWE were used to measure Young's modulus of flexor carpi radialis muscle with joint from 0° (at rest) to 50° flexion (passive stretch condition), with 10° interval. The neural (NC) and non-neural components i.e., elasticity component (EC) and viscosity component (VC) of the wrist joint were analyzed from a motorized mechanical device NeuroFlexor® (NF). Combining with a validated biomechanical model, the neural reflex and muscle stiffness contribution to the increased resistance can be estimated. MAS and Fugl-Meyer upper limb score were also measured to evaluate the spasticity and motor function of paretic upper limb. Young's modulus was significantly higher in the paretic side of flexor carpi radialis than that of the non-paretic side (*p* < 0.001) and it increased significantly from 0° to 50° of the paretic side (*p* < 0.001). NC, EC, and VC on the paretic side were higher than the non-paretic side (*p* < 0.05). There was moderate significant positive correlation between the Young's Modulus and EC (*r* = 0.565, *p* = 0.028) and VC (*r* = 0.645, *p* = 0.009) of the paretic forearm flexor muscle. Fugl-Meyer of the paretic forearm flexor has a moderate significant negative correlation with NC (*r* = −0.578, *p* = 0.024). No significant correlation between MAS and shear elastic modulus or NF components was observed. This study demonstrated the feasibility of combining SWE and NF as a non-invasive approach to assess spasticity of paretic muscle and joint in stroke clinics. The neural and non-neural components analysis as well as correlation findings of muscle stiffness of SWE might provide understanding of mechanism behind the neuromuscular alterations in stroke survivors and facilitate the design of suitable intervention for them.

## Introduction

Stroke is one of the major causes of long-term disability worldwide and leads to motor and sensory impairments on upper and lower extremity of survivors (1, 2). Spasticity, a neurological problem secondary to stroke, has a significant effect on skeletal muscle and affects motor function and quality of life (3, 4). Despite the high prevalence of spasticity in people with stroke, the underlying mechanism remains poorly understood due to the confusion in concept of spasticity. Alterations of spastic muscle might not simply due to chronic stimulation or disuse, and literatures showed that exaggerated reflexes and secondary changes in mechanical muscle properties may have a major role in spastic movement disorder (5). Understanding the mechanical and neurophysiological characteristics of spasticity may provide important clues to its intervention.

Previous studies involving a variety of methods studying joint and tissue mechanics as well as muscle morphology offered clear evidence to suggest that skeletal muscle tissue itself is altered under spastic conditions (6). The mechanical property of spastic muscle could be assessed by imaging technique such as ultrasound. Architectural parameters such as pennation angles, fascicle lengths, and muscle thickness assessed by ultrasound could quantitatively evaluate the morphological characteristics of the muscle tendon complex (7–9). Shortened muscle fascicle length and smaller physiological cross section area were observed in patients with stroke (7). However, muscle function is not only related to its morphology parameters but also the mechanical properties. Shear wave elastography (SWE) tracks the propagation of the shear wave through the muscle tissue using ultra-fast ultrasonic imaging and shear wave travels faster through stiffer tissues (10). It is a new approach to provide a real-time quantitative metrics of tissue material properties, including mechanical properties such as stiffness of skeletal muscles in upper and lower limbs (11–15). Koo and coworkers found SWE was significantly correlated with passive muscle stiffness in both animal models (16) and healthy subjects (17). Bouillard applied SWE and electromyography (EMG) to estimate individual muscle force of healthy subjects and found a significant linear correlation between shear elastic modulus and muscle force (18). These findings suggest that SWE may be a feasible way to quantify the inherent strain-stress behavior of muscle after neuromuscular disease. Published studies showed that shear wave speed and echo intensity were higher in the relaxed paretic limb than in the relaxed non-paretic limb of stroke survivors (19–21), multiple sclerosis (22), cerebral palsy (23–25), and Duchanne muscular dystrophin (26). However, previous studies of SWE on spastic muscle mainly focused on investigating relaxed muscles with a specific posture (19–21). A systematic assessment of muscle stiffness in relation to joint angle is needed to provide comprehensive information on muscle properties alterations after pathology and how they relate to the ability to conduct activities of daily living (ADL) such as feeding and dressing.

In clinics, physicians often examine the alterations in muscle stiffness by passively stretching the spastic limbs and palpating the spastic muscle. The outcomes of the assessments are subjected to the experience of the clinicians. The sub-optimal evaluation method adopted in clinics may due to the fact that quantitative assessment of spastic muscle-tendon-joint is rare. Spasticity, defined as hyper-resistance measured during passive rotation of a joint, is related to neural and non-neural factors (27–29). To separate and analyze individual components of this hyper-resistance would lead to better understanding of spasticity and further assist the selection of appropriate intervention (28). The NeuroFlexor (Aggro MedTech AB, Solna, Sweden) is a recently developed motorized instrument which can passively extend the wrist joint at controlled velocities (30). Based on the biomechanical modeling method, the recorded resistant force can be separated into three contributors to the measured resistant force: neural component (NC), elasticity component (EC), and viscosity component (VC) (31, 32). It was found that the NC and EC increased in paretic side compared to that from non-paretic side in stroke survivors (28.30). In addition, it was reported that the NC decreased significantly after injections of Botulinum toxin type A, but the passive components of EC and VC did not change overtime (31). The NeuroFlexor (NF) has been also used to evaluate neural and non-neural contributions to the wrist resistance in patients with Parkinson's disease and cerebral palsy (30–33). The validity of the NF-method has been demonstrated through the strong correlation between the NC and the EMG activity of flexor carpi radialis, both across all subjects and between-subject during the nerve block test in the stroke survivors (31, 34). However, there is still controversial opinion on whether the neural factor from central nervous system or the mechanical factor of musculotendon system is a primary contributor to spasticity (35). In addition, there is limited information on whether the non-neural components of resistance measured by the NF-method are clinically relevant. For instance, it is unclear that whether non-neural components are associated with intrinsic muscle mechanical properties.

The aim of the present study was to investigate the feasibility and applications of combining SWE and NF to quantitatively assess musculoskeletal properties alterations in spastic muscle joint post-stroke. Understanding the delicate variations of muscles properties during passive movement, and the neural and non-neural components that contribute to joint resistance might facilitate the development of targeted therapeutic regime to address spasticity for motor function improvement.

## Materials and Methods

### Study Design

This study was a cross-sectional study on spasticity of the wrist flexors. We aimed to measure passive stiffness of spastic wrist flexors on the paretic side and compared to the non-paretic side in stroke survivors. This study was performed at Department of Rehabilitation of the First Affiliated Hospital, Sun Yat-sen University, China. This study was approved by the Ethics Committee of the Hospital. All procedures were conducted according to the Declaration of Helsinki and all subjects provided written consent before the experiments.

### Participants

Inclusion criteria of stroke patients were patients who: (1) suffered the first occurrence of stroke (as confirmed by computed tomography or magnetic resonance imaging) that resulted in unilateral hemiparesis; (2) at least 1 month post-stroke; (3) had passive range of motion of wrist joint from −20° palmar flexion to 50° dorsiflexion; and (4) scored ≥ 1 on the Modified Ashworth Scale (MAS) (36). This study was approved by the Ethics Committee of the First Affiliated Hospital, Sun Yat-sen University (ethics number: [2017].143). All subjects were provided written consent before the experiments. Study procedures were conducted according to the Declaration of Helsinki. The MAS [the protocol from Bohannon and Smith (36)], and upper limb Fugl-Meyer Assessment (FMA) of recruited stroke subjects were evaluated by a licensed physiotherapist who was blind to the experimental results.

### Procedures and Devices

**Figure 1:**
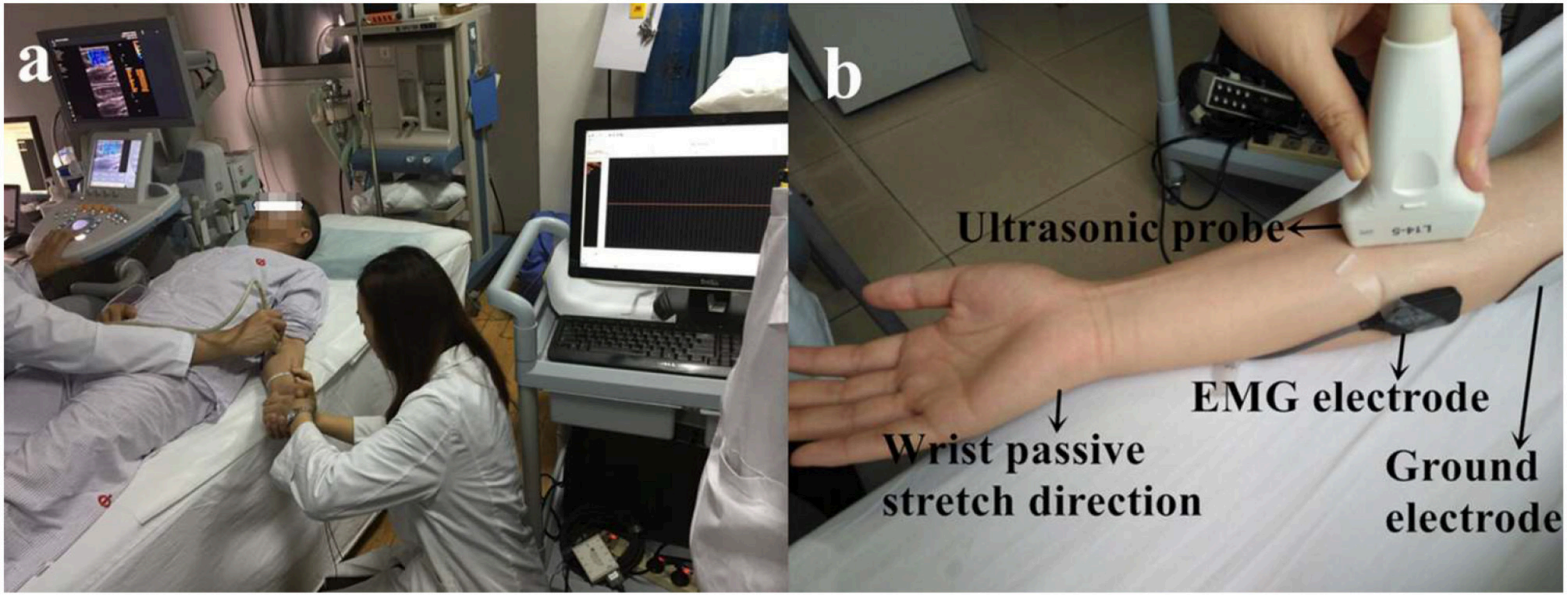
The experimental setup **(a)** and detailed position of the probe and EMG electrode on the muscle **(b)**. Consent was obtained from the individual for the publication of this image.

Baseline data and anthropometric characteristics of the stroke survivors were collected before the experiment began, including age, gender, paretic side, time since stroke, and body weight. The ultrasound measurements were followed the standard procedures of the Department of Medical Ultrasonics and all the ultrasound images had been taken by an experienced sonographer (ZW). SWE ultrasound image was captured using an AixPlorer ultrasonic scanner (Supersonic Imagine, Aix-en-Provence, France). The probe (L10-2, 4-15 MHz, SuperLinear 15-4, Vermon, Tours, France) was placed carefully over the flexor carpi radialis muscle belly of the wrist joint. Subjects were asked to lay in supine position comfortably and to leave their upper limb resting on the mat. The ultrasound transducer was held on the subject's limb by the operator. Ultrasound coupling gel was applied to enhance conduction between the ultrasound probe and skin surface. Care was taken during ultrasound scanning when positioning the probe so that data were collected from a similar location within the muscle belly for all collections with wrist angles ranging from 0° palmar flexion to 50° dorsal flexion (0°, 10°, 20°, 30°, 40°, 50° of wrist joint and set randomly) (Figure 1a). Subjects were asked to remain as relaxed as possible during shear modulus measures (37). To confirm the muscle did not have active contraction movement during passive stretch, EMG sensor (Bagnoli-8, Delsys Inc., Boston, USA) was placed on flexor carpi ulnaris muscle longitudinally with respect to the underlying muscle fiber arrangement and distal to the probe. A representative flexor carpi ulnaris EMG activity was also obtained. Before the electrode placement, the area around the tested muscles were shaved and cleaned with alcohol. Surgical tape was used as appropriate around the electrode and amplifier to obtain EMG signals. EMG signals were digitized at a sampling rate of 2,000 Hz (by NI PCI-6220 system, USA) and displayed in real-time to ensure that there was no active muscle contraction and the subject was at resting condition during the SWE data acquisition (Figure 1b).

**Figure 2:**
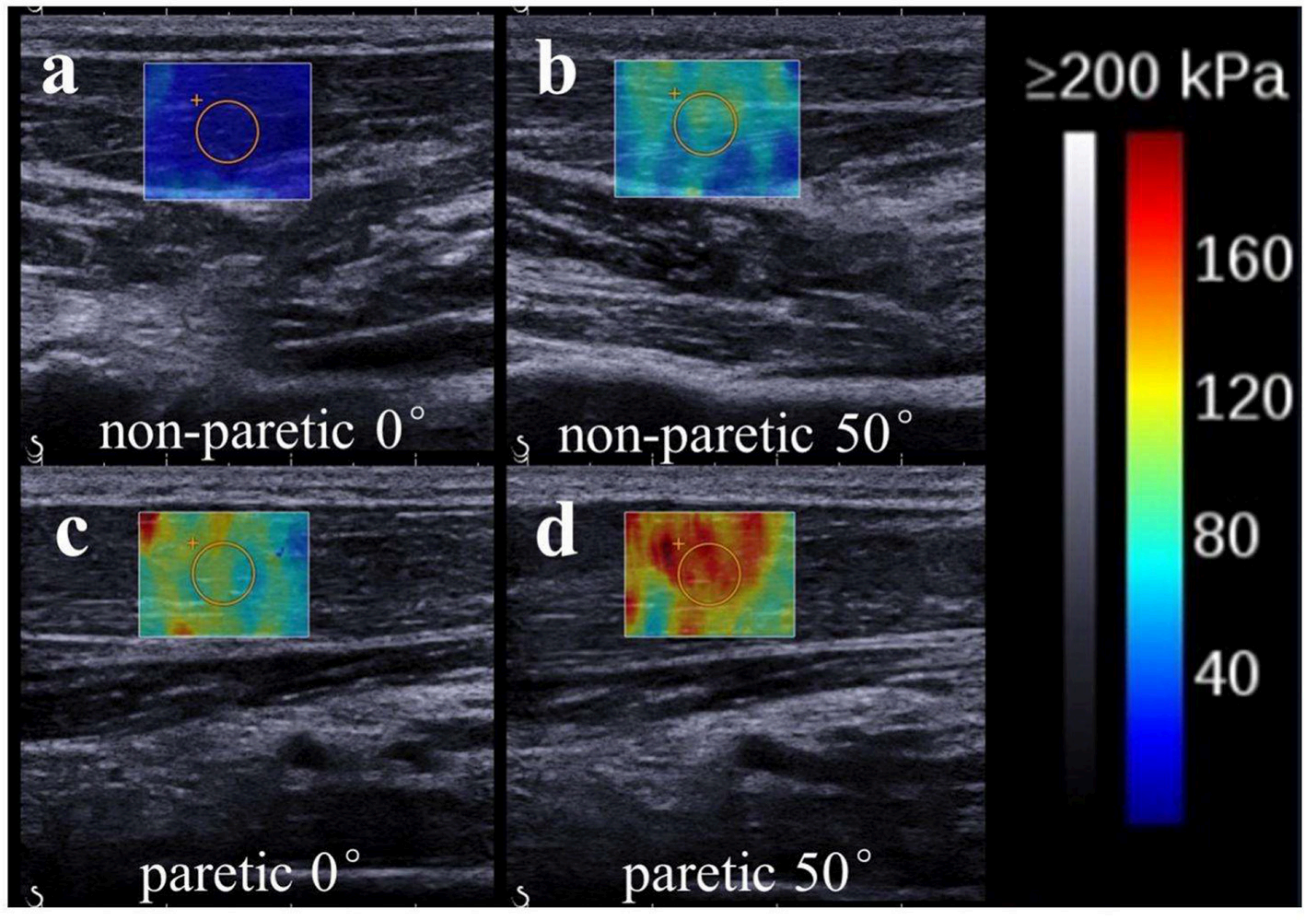
Representative ultrasound images (SWE ultrasound map superimposed on the B-mode image) of the non-paretic (**a**:0°; **b**:50°) and paretic (**c**:0°; **d**:50°) flexor carpi radialis. The value of shear wave modulus is greater in the paretic limb. We found the non-paretic muscle has a lower value of shear elastic modulus than the paretic muscle at the same wrist angle. Meanwhile, non-paretic and paretic muscle both has a lower value of shear elastic modulus when wrist was held at 0° than wrist was passive stretched at 50°.

The ultrasound system was set in supersonic shear imaging (SSI) mode with musculoskeletal preset. Ultrasonic B-mode images and shear wave modulus were collected simultaneously. The technical details of this technology used in the musculoskeletal system have been described previously (11, 38, 39). In brief, shear waves are formed within the tissue as they interact with conventional ultrasound waves produced by the transducer. An ultrafast ultrasound imaging sequence is then performed to determine shear wave velocity (Vs) along the principal axis of the probe. The shear elastic modulus (μ) is a biomechanical measurement of hardness reported in kilopascal (kPa). It is calculated by multiplying the squared Vs by the muscle mass density ρ (1,000 kg/m^3^): μ = ρ Vs^2^. (40, 41). The shear elastic modulus was calculated in the region of interest (ROI) with Q-Box function, which was set to 10^*^10 mm^2^, and the depth was set to ~0.5–1.0 cm. The two-dimensional gray-scale and elastic images were simultaneously observed with double real-time imaging (Figure 2). Shear wave images were collected with three trials of five images collected at each joint angle (Figure 2). The values of shear elastic modulus of each angle were then averaged.

**Figure 3:**
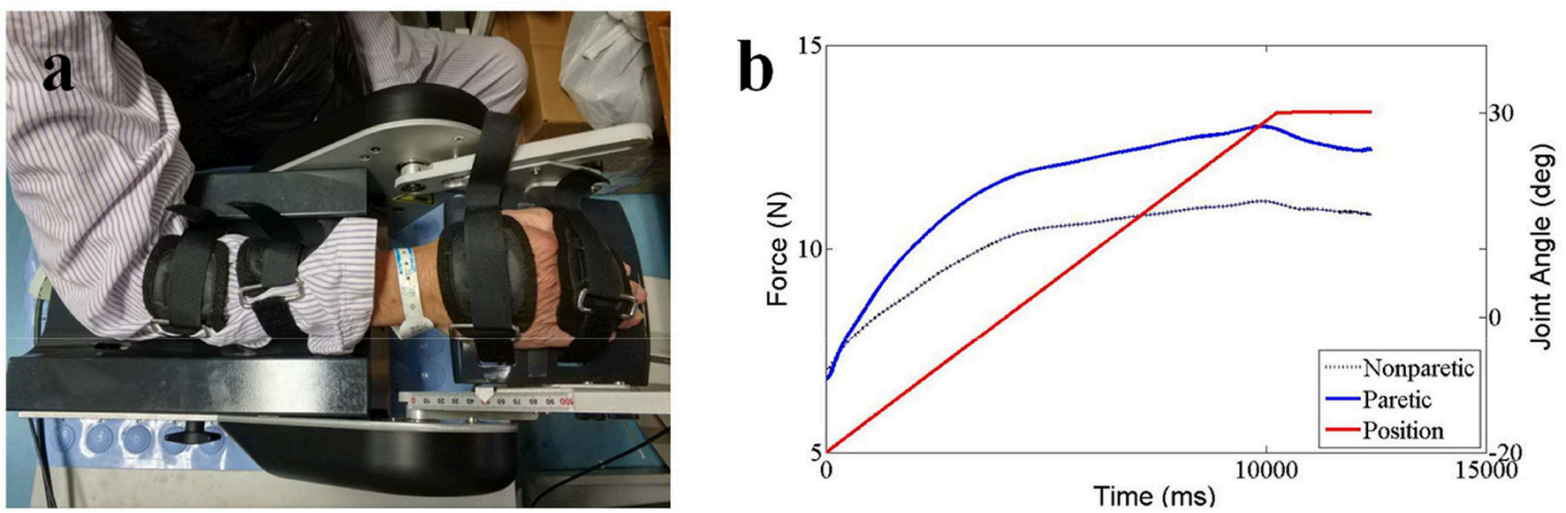
NeuroFlexor Method testing setup **(a)** and recording joint angle and resistance torque **(b)**.

For the NeuroFlexor (NF) measurement, subjects were seated next to the instrument comfortably and rested their hands on the platform. The shoulder was placed in ~45° abduction, elbow in 90° flexion, and the forearm pronated (31). The NeuroFlexor instrument produced passive movement at constant speeds and record the passive resisting forces from corresponding joint in real time by the force sensor under the moveable platform (Figure 3a). With the metacarpophalangeal joints in slight flexion and the fingers fully extended, both hands and arms were fastened using non-elastic velcro straps in order to ensure that movement could only occur at the wrist joint (30). During the joint movement, time, angle, and resisting forces were recorded simultaneously (Figure 3b). Several trails were given to subjects to become accustomed to the device. The subject was asked to stay relaxed during testing and no active movement of limbs or head during the passive movements was instructed. The NF instrument ran 7 times at slow mode of 5°/s (including 2 times without hand and 5 times with hand) and 12 times at fast mode of 236°/s (including 2 times without hand and 10 times with hand). The range of wrist movement was 50° with a starting position at −20° palmar flexion and an end position at 30° extension. There were 10 s interval between each fast trial (32).

Three components were calculated based on a biomechanical model that had been shown to be valid and reliable for the measurement of spasticity in stroke patients (28, 30, 31, 34). Briefly, NC represents the contribution from spasticity. It is estimated at maximal extension at the end of the passive movement (30° wrist extension) by subtracting the EC and VC from the total force. EC represents the length-dependent resistant force and is recorded 1 s after the end (30° wrist extension) of the slow stretching movement (5°/s) to minimizing possible contribution from stretch reflexes. VC, represents the velocity-dependent resistant force, is calculated at the end of the passive movement (30° wrist extension). It is approximated to be 20% of the first peak in the fast movement resisting the acceleration of the hand which depends on the mass of hand and moveable platform and the acceleration (32). All of the three components represent the statement of the muscles stretched at 30° wrist extension.

### Statistical Analysis

Descriptive statistics (mean values and standard deviation) were calculated for all dependent variables. The statistical analyses were conducted using SPSS 19 (IBM, United States). Shear elastic modulus values were analyzed with a repeated-measures two-way analysis of variance (ANOVA) (between group factor: paretic and non-paretic; within group factor joint angle: 0°~50°). The results were further compared using a Bonferroni *post hoc* test. Three components of NF (NC, EC, and VC) were also compared between paretic and non-paretic side with paired *t*-test, respectively. The correlations between the SWE data, Fugl-Meyer score, and each components of NF were determined using Pearson correlations. Spearman's correlations were used to examine the relationships between MAS score and the SWE data or each component of NF. The significant level of all statistical analyses was set at 0.05.

## Results

Fifteen stroke survivors (12 males, 3 females, mean ± SD, age: 54 ± 8 years, body weight: 61 ± 6 kg, height: 164 ± 7 cm) participated in the current study. The clinical characteristics and functional level of the sample population were summarized in Table 1. Typical images of SWE of wrist flexor in stroke survivor are shown in Figure 2. There was no significant difference in the mean value of share elastic modulus when different ROI's size was selected.

**Table 1:** Background data of the stroke survivors.

**Subject**	**Age (year range)**	**Duration (month)**	**Paretic hemisphere**	**Clinical scales**	
				**FMA**	**MAS**
1	50–55	4	L	12	1+
2	60–65	2	R	6	2
3	50–55	1	L	10	1
4	35–40	12	R	37	1+
5	50–55	4	L	14	2
6	40–45	1	L	14	1
7	45–50	5	L	26	1
8	55–60	1	L	26	1
9	50–55	12	R	55	1+
10	65–70	2	R	12	1+
11	50–55	24	L	57	2
12	60–65	2	R	53	1+
13	60–65	12	L	52	1+
14	45–50	2	R	30	2
15	60–65	8	L	6	2

*R, right = 6; L, left = 9; FMA, Fugl-Meyer assessment scale of the motor function in paretic upper-extremity; MAS, Modified Ashworth Score*.

**Figure 4:**
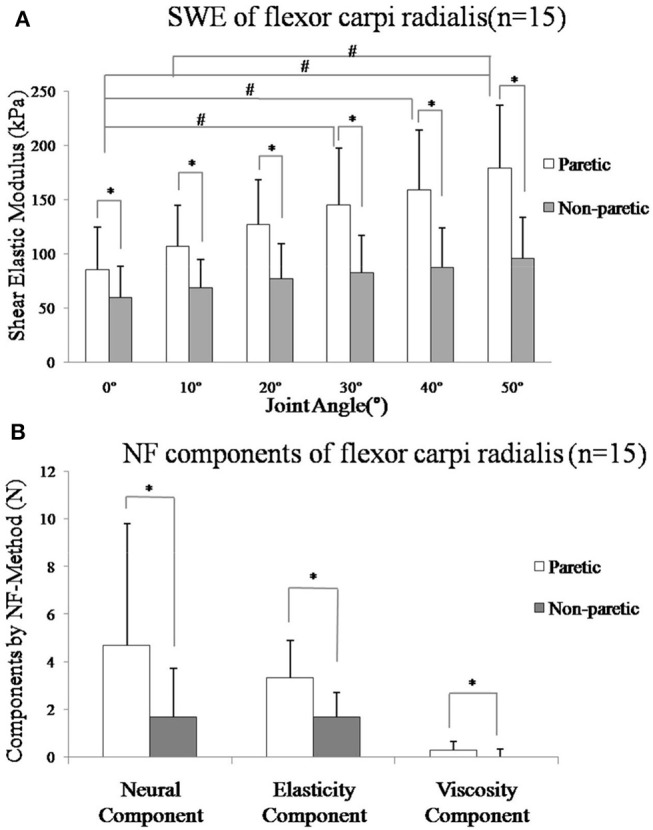
**(A)** Mean value of shear elastic modulus and standard deviation of the paretic (p: white bars) and non-paretic (n: gray bars) flexor carpi radialis muscle from all subjects in different angle. All subjects (*n* = 15) SME was greater in the paretic muscle in each angle (*p* < 0.001). A significant increase was found in paretic side from 0° to 50° (*p* < 0.001, *post-hoc* tests on the difference angle between 0° and 30°, *p* = 0.016, 0° and 40°, *p* = 0.001, 0° and 50°, *p* < 0.001, 10° and 50°, *p* = 0.001), but no significant in non-paretic side (*p* = 0.05). **(B)** Mean value of components by NeuroFlexor method and standard deviation of the paretic (p: white bars) and non-paretic (n: gray bars) forearm flexor muscle from all subjects. The mean value of NC (Neural Component), EC (Elasticity Component), and VC (viscosity component) of all subjects (*n* = 15) was greater in the paretic side (*p* < 0.05). *Represents statistically significant between the groups, ^#^Represents statistically significant with different angles within the group.

As shown in Figure 4A, two-way ANOVA results showed that shear elastic modulus were significantly higher in paretic side than non-paretic side (*p* < 0.001), but interaction of two factors (side and angle) was also statistically significant (*p* = 0.044). A significant increase was found in paretic side from 0° to 50° using a one-way ANOVA (*p* < 0.001, *post hoc* tests on the difference angle between 0° and 30°, *p* = 0.016, 0° and 40°, *p* = 0.001, 0° and 50°, *p* < 0.001, 10° and 50°, *p* = 0.001), but no significant in non-paretic side (*p* = 0.05). Mean value of shear elastic modulus was significantly higher in paretic side than non-paretic side at each angle, respectively (*t*-test, *p* < 0.001).

As shown in Figure 4B, the mean values of NC, EC, and VC of all subjects (*n* = 15) on the paretic side (NCp = 4.67 ± 5.12, ECp = 3.33 ± 1.56, VCp = 0.27 ± 0.38) were higher than the non-paretic side (NCn = 1.69 ± 2.02, ECn = 1.70 ± 0.99, VCn = 0.01 ± 0.30), respectively (*p* < 0.05).

**Figure 5:**
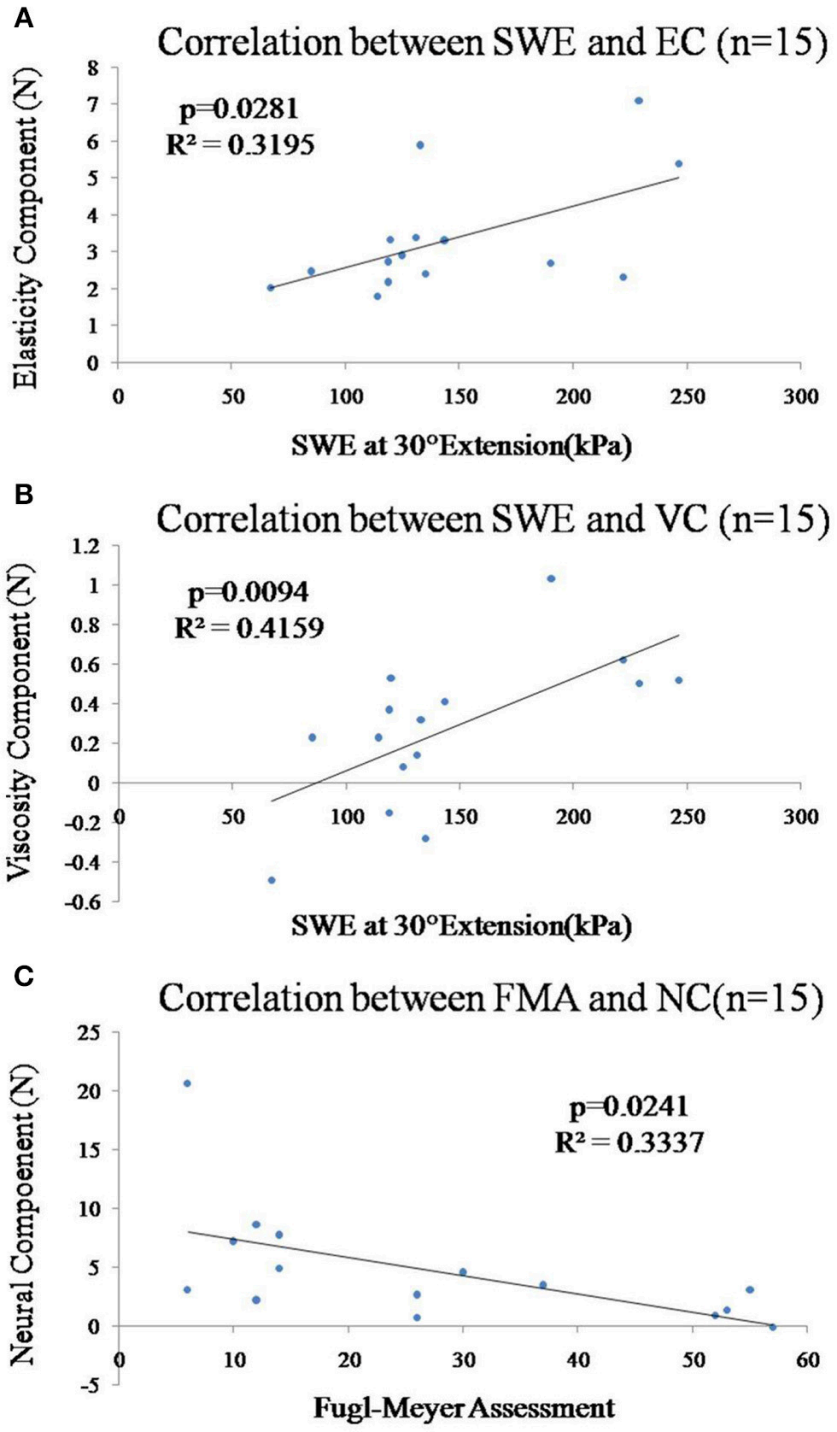
**(A)** Correlation between the shear elastic modulus at 30° wrist extension and EC of the paretic forearm flexor muscle was significant (*p* = 0.028). **(B)** Correlation between the shear elastic modulus at 30° wrist extension and VC of the paretic forearm flexor muscle was significant (*p* = 0.009). **(C)** Correlation between the Fugl-Meyer score and NC of the paretic forearm flexor muscles was significant (*p* = 0.024).

There was moderate positive correlation between the shear elastic modulus and EC (Figure 5A, *r* = 0.565, *p* = 0.028) or VC (Figure 5B, *r* = 0.645, *p* = 0.010) of the paretic forearm flexor muscle. However, there was no significant correlation between the shear elastic modulus and NC (*p* > 0.05).

In Figure 5C, there was significant moderate correlation between the Fugl-Meyer score and NC (Figure 5C, *r* = −0.578, *p* = 0.024) of the paretic forearm flexor muscle. However, there was no significant correlation between Fugl-Meyer score and VC/EC (*p* > 0.05) or mean value of shear elastic modulus (*p* > 0.05). All the correlations between the MAS and shear elastic modulus or components of NF (NC, EC, VC) of the paretic forearm flexor muscle were not significant (*p* > 0.05).

## Discussion

This study investigated the feasibility of combining SWE technique and biomechanical model to objectively and quantitatively assess alterations of paretic muscle stiffness with joint angles in stroke survivors. Significant correlation was revealed between the shear elastic modulus and EC and VC of Neuroflexor method. In addition, the SWE data and NF components data were partially correlated with clinical scales.

### Shear Elastic Modulus and Muscle Stiffness

The shear elastic modulus of the paretic flexor carpi radialis muscle was significantly greater on the paretic side than the non-paretic side (Figure 4A). This finding is in line with previous study on biceps brachii of stroke survivors using SWE (19, 20). Researchers investigated the feasibility of elastography to assess gastrocnemius muscle stiffness in post-stroke survivors (10, 21). It was found that elastic modulus measured on the paretic side was significantly larger than that on non-paretic side. As the wrist was passively stretched from 0° palmar flexion to 50° extension, shear elastic modulus increased significantly accordingly in the paretic flexor carpi radialis muscle. Similar findings were reported by Lee et al. in persons with cerebral palsy, that shear elastic wave speed increased as torque, ankle angle, and fascicle strain increased (25). The mechanism behind elastic modulus changes in spastic muscle is still under discussion. One possible explanation might be related to muscle structural alterations after stroke. In literature, shortened muscle fascicle length at upper (7) and lower extremities (8) has been observed in stroke survivors using B mode ultrasound image. These literature results suggest that altered muscle morphology of the paretic muscle may contribute to abnormal muscle elastic property during passive stretch (42). Another possible reason of the increased elastic modulus on the paretic side is muscle composition changes. These changes include increased perimysium and myofibril in collagen, titin and extracellular matrix accumulated abnormally in intracellular proteins, and increased fat (43–45). Muscle compositions contribute to muscle elasticity and previous studies on rat models of spinal cord injury also revealed that spastic muscle had extracellular connective tissue modifications during recovery. Therefore, those pathological muscle structure and compositions alterations might cause an increased in Young's modulus as quantified by SWE which in turn contribute to higher stiffness of paretic joint as measured by the NF in the current study. However, we cannot conclude this without further investigation. Future study combined with different techniques measuring muscle inherit properties such as biopsy and motor unit number estimation (MUNE) would be helpful to validate the findings and gain better understanding of paretic muscle functional changes after stroke.

### Components Analysis From Biomechanical Model

NC, EC, and VC estimated by the NF method were significantly larger on the paretic side, which led to a higher total resistant force (Figure 4B). This finding was consistent with early studies (30–32). Wang et al. (28) used the NF method on stroke patients and found EC was the largest contributor and VC was the least contributor to the total resistant torque in people with lower level of spasticity. The increase in NC might be related to the hyperactive reflex resulted from abnormal synaptic input-output relationship, and altered intrinsic properties of the motor neurons after stroke (28–46). There is no stretch reflex in static state or during very slow stretching movement (47). We found moderate correlations between the SWE and EC as well as with VC. The EC and VC components quantify the overall elasticity and viscosity of the muscle-tendon-joint system (34), while SWE quantify the muscle elasticity (13, 14, 20, 21). Our findings indicated that the two methods do not measure identical parameters, but they are complementary to each other and both helpful in quantifying spasticity reciprocally. The spasticity which reflects a phenomenon of abnormal recovery is only one of the factors in motor dysfunction after stroke (48, 49). As previously described, EC is a length-dependent resisting force that increases as the muscles and tendons are stretched. In literature, shortened muscle fascicle length has been observed in post-stroke survivors (7, 43, 44). There are also other perspectives such as muscle fibers damage led to an increased myoplasmic calcium (50), resulting in an increased number of residual cross-bridges between myosin heads and actin and leading to greater passive stiffness (51). Therefore, the correlation between SWE and NF-method not only offer alternative ways to quantify neural and non-neural related contributors to the spasticity but also provides clinical insights for targeting suitable recovery strategies of muscle tissue. These findings suggest that intervention of spasticity should not only focus on neural component and should include strategies to improve muscle mechanical properties.

### Clinical Correlations

In the current study, we found that Fugl-Meyer had a significant correlation with NC of NeuroFlexor but no significant relationship between Fugl-Meyer and SWE data. Fugl-Meyer is commonly used to assess limbs motor function that can be influenced by factors such as neural muscle activity, muscle strength, muscle tension, and body physiological effect (49, 52). Compared to the SWE, the measurement of NF estimates the overall joint properties, which reflects the joint function better as indicated by stronger correlation with Fugl-Meyer score (28, 48). Even though there is correlation observed between Fugl-Meyer and NF method parameters, no firm conclusion could be drawn on whether the NF components could be used as a predictor for motor function without further investigation. We found that MAS scores of spastic muscles showed no significant correlation with SWE or NF measurements, which was in line with previous study (31). Contrary findings were reported by Kesikburun et al. (21), who revealed a significantly correlation between MAS and shear elastic modulus value on gastrocnemius of stroke survivors, and by Lindberg PG, who found NC correlated well with the MAS score (34). There is no gold standard to measure spasticity in a clinical setting (31). Many researchers considered the reliability of MAS to be poor to moderate as it is scored subjectively by the examiner which is likely to be influenced by placebo effect (53). Factors like raters' experience, the velocity and range of motion, repeated stretching, and statistical regression all have had a role in poor validity and reliability. Especially, the factor rater appeared to be highly associated with MAS, and some researchers even consider the validity and reliability of the MAS is insufficient to be used as a measure of spasticity (54, 55). Therefore, the SWE and NF measurements could be a better alternative as quantitative methods to evaluate individual contributors to the spasticity in stroke survivors.

### Limitations

There are some limitations in the current study which limit the interpretation and generalizability of the data. First, all of the SWE and joint force data were measured under passive stretch condition. Thus, muscle properties during voluntary contraction could not be quantified. In addition, the sample size of the current study may be considered as small. We could not divide the sample population into acute, subacute, or chronic group and the current sample population included subjects who had mild to moderate spasticity. Future study should include large sample size with patients who have a range of level of muscle stiffness at different stages of stroke. One more limitation of the study is using the Bohannon-Smith MAS for grading spasticity (36). Previous research shows high inter-rater or intra-rater reliability of the Modified Modified Ashworth Scale (MMAS). The MMAS score with the clear definitions and hierarchical relationship of the grades of 1 and 2 have an ordinal relationship by omitting the grade “1+” and redefining grade “2,” that would be more valid for grading the lower grades of spasticity (56, 57). This was a preliminary study aimed to demonstrate the feasibility of using SWE in combination of NF to document muscle changes passively with joint angles. In future study, other methods such as EMG recording during active contraction and muscle tone measurement (58) might be useful to determine the muscle properties changes after stroke in dynamic condition.

## Conclusion

In conclusion, we have applied SWE combined with biomechanical model from Neuroflexor method as non-invasive approaches to assess spasticity of paretic muscle in people with stroke. The correlation analysis showed non-neural mechanical factors of the muscle significantly related with the Young's modulus during wrist passive extension. The combined methods could help to better quantify the non-neural factors secondary to spasticity e.g., increased joint and muscle stiffness, and provide valuable insights in design of suitable intervention program for persons after stroke.

## Ethics Statement

This study was approved by the Ethics Committee of the First Affiliated Hospital, Sun Yat-sen University (ethics number: [2017].143). All subjects were provided written consent before the experiments. Study procedures were conducted according to the Declaration of Helsinki.

## Author Contributions

YL, LL, and DH conceived and designed the study. YL, ZW, and RB performed the experiments. YL and RW wrote the paper. RW, XX, and WL made a contribution to experiments. RW, XX, WL, DH, and LL reviewed and edited the manuscript. All authors had read and approved the manuscript.

### Conflict of Interest Statement

The authors declare that the research was conducted in the absence of any commercial or financial relationships that could be construed as a potential conflict of interest. The handling editor declared a past supervisory role and past co-authorship with one of the authors LL.
